# Prediction of *S*-Glutathionylation Sites Based on Protein Sequences

**DOI:** 10.1371/journal.pone.0055512

**Published:** 2013-02-13

**Authors:** Chenglei Sun, Zheng-Zheng Shi, Xiaobo Zhou, Luonan Chen, Xing-Ming Zhao

**Affiliations:** 1 Institute of Systems Biology, Shanghai University, Shanghai, China; 2 School of Communication and Information Engineering, Shanghai University, Shanghai, China; 3 Department of Computer Science, School of Electronics and Information Engineering, Tongji University, Shanghai, China; 4 Key Laboratory of Systems Biology, Shanghai Institutes for Biological Sciences, Chinese Academy of Sciences, Shanghai, China; 5 Department of Radiology, The Methodist Hospital Research Institute, Weill Medical College of Cornell University, Houston, Texas, United States of America; St. Georges University of London, United Kingdom

## Abstract

*S*-glutathionylation, the reversible formation of mixed disulfides between glutathione(GSH) and cysteine residues in proteins, is a specific form of post-translational modification that plays important roles in various biological processes, including signal transduction, redox homeostasis, and metabolism inside cells. Experimentally identifying *S*-glutathionylation sites is labor-intensive and time consuming, whereas bioinformatics methods provide an alternative way to this problem by predicting *S*-glutathionylation sites *in silico*. The bioinformatics approaches give not only candidate sites for further experimental verification but also bio-chemical insights into the mechanism of *S*-glutathionylation. In this paper, we firstly collect experimentally determined *S*-glutathionylated proteins and their corresponding modification sites from the literature, and then propose a new method for predicting *S*-glutathionylation sites by employing machine learning methods based on protein sequence data. Promising results are obtained by our method with an AUC (area under ROC curve) score of 0.879 in 5-fold cross-validation, which demonstrates the predictive power of our proposed method. The datasets used in this work are available at http://csb.shu.edu.cn/SGDB.

## Introduction

*S*-Glutathionylation, the reversible formation of protein mixed disulfides between cysteine residues and glutathione(GSH), is an important form of posttranslational modification that occurs during oxidative stress as well as under normal physiological conditions [1]–[4]. When exposed to reactive oxygen/nitrogen species (ROS/RNS), *S*-glutathionylation is induced along with other forms of thiol oxidation (e.g., sulfenic, sulfinic, or sulfonic acids). Since *S*-glutathionylation is a reversible modification, likely catalyzed by glutaredoxins (Grx), it can serve to protect critical protein thiols from irreversible oxidation and inactivation. Operationalized proteins also serve as a storage form of GSH under oxidative stress, where the oxidized glutathione (GSSG) is otherwise exported from the cell. It has been recently hypothesized that glutathionylation is a general mechanism for protein function and cell signaling, similar to the protein phosphorylation system mediated by kinases and phosphatases [1]–[4]. *S*-glutathionylation has been shown to cause either inhibition or activation of a number of proteins. Generally, these proteins fall into the following functional categories: metabolism and energy, cell signaling (particularly kinases and phosphatases), calcium homeostasis, redox homeostasis, protein folding and degradation, and the cytoskeleton (including actin, tubulin, and vimentin) [2], [4]. Recently, a growing body of evidence has linked protein *S*-glutathionylation with pathogenesis of many human diseases including metabolic disorders, cardiovascular diseases, lung diseases, cancer, and neurodegenerative diseases [1]–[4].

Recently, various experimental methods were used to identify *S*-glutathionylation proteins, including spectrophotometric assays, high performance liquid chromatography assays, liquid chromatography-mass spectrometry, etc [5], [6]. Until the writing of this paper, more than 100 *S*-glutathionylation proteins have been identified, but only a minority of them have their *S*-glutathionylation sites identified. Experimentally identifying *S*-glutathionylation sites is a labor-intensive and time consuming procedure. Despite some bioinformatics approaches have been proposed to predict catalytic redox-active cysteine [7], [8] and the disulfide bonding state of cysteine [9]–[11], they are not able to predict *S*-glutathionylation sites due to the scarcity of *S*-glutathionylation sites and protein structure data.

In this paper, we present a bioinformatics framework to predict *S*-glutathionylation sites by employing machine learning methods based on protein sequences. Despite the incompleteness of protein structures, it is found that the flanking residues of a functional site in sequence can give the context information of this residue in protein structure and are widely used to predict post-translational sites [12], [13]. Therefore, in this paper, we aim to explore different sequence descriptors and develop a framework by employing machine learning methods to predict *S*-glutathionylation sites based on protein sequences. Firstly, all the *S*-glutathionylation proteins and their corresponding modification sites are manually collected from the literature. Secondly, a series of classifiers are built to predict *S*-glutathionylation sites based on support vector machines (SVMs). Especially, different features are extracted from protein sequences for prediction of *S*-glutathionylation sites. Results obtained in 5-fold cross-validation demonstrate the effectiveness of our method with an AUC score of 0.879. To our best knowledge, this is the first work that use bioinformatics methods to predict *S*-glutathionylation sites, which can provide biologists putative *S*-glutathionylation sites for future experimental verification.

## Results

In this work, each cysteine residue and its flanking sequence was represented as a feature vector (see Methods). In this way, the supervised classifier can be constructed to predict *S*-glutathionylation sites. To validate our proposed method, 5-fold cross-validation was utilized for our collected *S*-glutathionylation site dataset with following indexes.
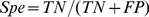
(1)

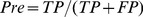
(2)

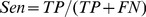
(3)

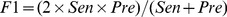
(4)

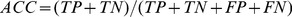
(5)

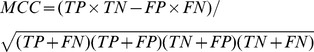
(6)where TP denotes number of true positives, TN denotes number of true negatives, FP denotes number of false positives, and FN denotes number of false negatives.

**Figure 1:**
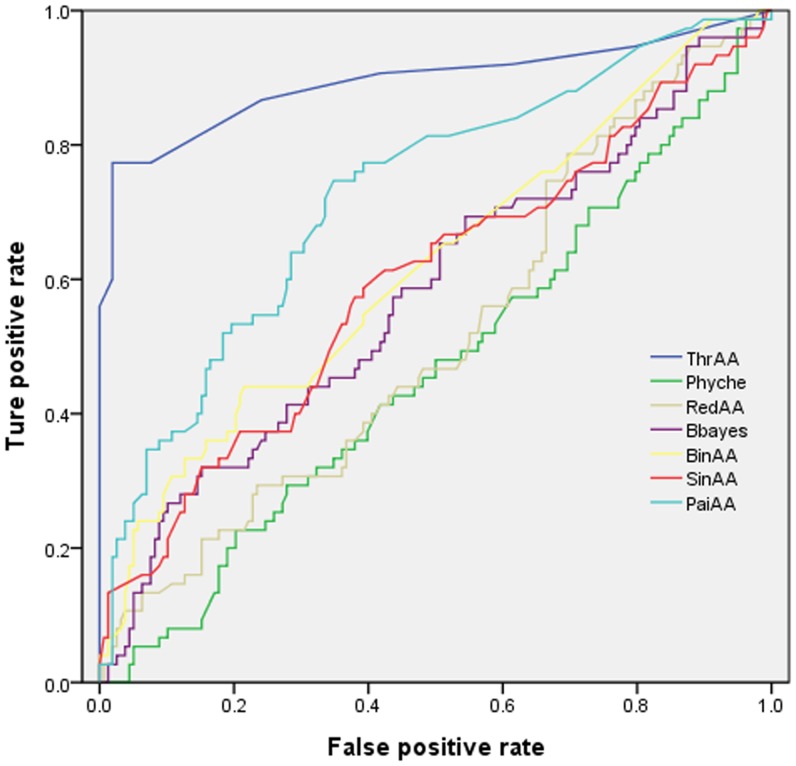
The ROC curves for different classifiers based on different feature extraction approaches. ThrAA: triplet amino acid composition; Phyche:10 physicochemical properties; RedAA: reduced amino acid composition; Bbayes: Bi-profile Bayes; BinAA: binary encoding amino acid; SinAA: amino acid composition; PaiAA: pair amino acid composition.

With the feature vectors available, for each feature extraction method, a classifier was constructed. Table 1 shows the results obtained by the classifiers trained with eight different sequence descriptors, where a simple feature selection approach based on Student's 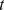-test was employed to pick optimal features (see Methods). From the results, we can see that multiple amino acid composition generally perform better than single amino acid composition. The best results were obtained by amino acid triplet (ThrAA) compositions, which is not surprising considering that the amino acid triplet can better describe the spatial neighborhood information so that it can provide structural and functional information of cysteine residue and provide clues to *S*-glutathionylation. In particular, the results obtained by reduced amino acid triplet prove that the amino acid triplet provides spatial context information of cysteine without considering the specific direction of amino acid triplet. The good performance of binary amino acid profile may attribute to its position specific information on amino acids, while other descriptors fail to describe the neighborhood information of cysteine and therefore lead to poor results. Furthermore, Figure 1 shows the ROC curves for different methods, which confirm again that amino acid triplet performs best. Hereafter, the method based on reduced amino acid triplet composition was used to predict new *S*-glutathionylation sites.

**Table 1:** 5-fold cross-validation results by SVM classifiers using different amino acid sequence encoding schemes.

Schemes	D	AUC	Spe	Pre	Sen	F1	MCC	ACC
10 physicochemical properties	10(3)	0.548	0.994	0.888	0.102	0.182	0.237	0.706
Reduced amino acid composition	10(6)	0.522	0.985	0.651	0.058	0.107	0.121	0.686
Bi-profile Bayes	30(18)	0.555	0.781	0.419	0.333	0.371	0.122	0.636
Binary amino acid profile	300(12)	0.668	0.867	0.626	0.469	0.536	0.366	0.738
amino acid composition	20(3)	0.554	0.892	0.487	0.217	0.300	0.144	0.674
pair amino acid composition	400(21)	0.721	0.890	0.632	0.397	0.488	0.334	0.731
amino acid triple composition	8000(76)	0.851	0.993	0.979	0.707	0.821	0.774	0.901
Reduced amino acid triple composition	4200(71)	0.879	0.993	0.981	0.773	**0.865**	**0.823**	**0.922**

D: dimensionality; AUC: area under ROC curve; Spe: specificity; Pre: precision; Sen: sensitivity; F1: F-measure; MCC: Matthews correlation coefficient; ACC: accuracy; the numbers in parenthesis denote the dimensionality of features selected by 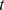-test.

**Figure 2:**
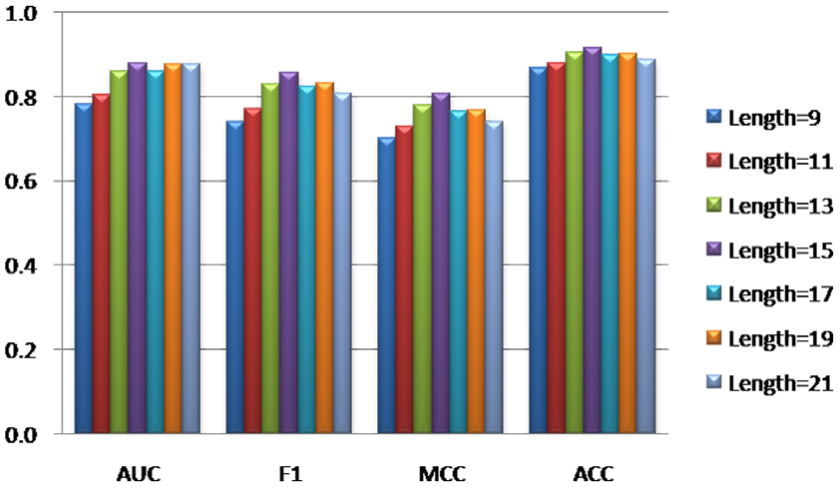
The effects of different window sizes on SVM performance.

In literature, different window sizes has been used to predict post-translational modification sites. For example, the upstream/downstream seven residues of phosphorylation site were considered (i.e. the window size is15) in GPS [14], while the window size was set to 13 in Musite [15] that is also a tool for predicting phosphorylation sites. Therefore, we investigated the effects of different window sizes on the performance of classifiers. Here, we considered window sizes ranging from 9 to 21. Figure 2 summarizes the results obtained with different window sizes, from which we can see that window size of 15 performs best according to 4 different indicators, i.e. AUC, F1, MCC and ACC. The detailed results can be found in Table S1. Therefore, window size of 15 was used as the optimal window size in this paper.

In constructing SVM classifier, there are generally three types of kernels that can be used, including linear kernel, polynomial kernel and radial basis function kernel (i.e. Gaussian kernel). In this work, we compared the performances of different kernels. Table 2 shows the results obtained by SVM classifiers with distinct kernels, where Gaussian kernel was found to perform best. Moreover, we compared SVM classifier against two other popular classifiers: 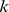-nearest neighbor and random forest classifiers, where the results were obtained based on the top ranked features for each classifier. In 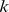-nearest neighbor classifier, the optimized value of 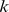 was set to 1. The results shown in Table 3 demonstrate that SVM classifier outperforms the two other ones. Therefore, SVM classifier with Gaussian kernel was adopted for future predictions in this paper.

**Table 2:** Comparison of the performance of three kernel functions of SVM.

Schemes	AUC	Spe	Pre	Sen	F1	MCC	ACC
linear kernel function	0.812	0.999	0.994	0.448	0.618	0.593	0.821
polynomial kernel function	0.819	1.000	1.000	0.307	0.470	0.481	0.777
radial basis kernel function	0.879	0.993	0.981	0.773	**0.865**	**0.823**	**0.922**

radial basis kernel function: Gaussian kernel; AUC: area under ROC curve; Spe: specificity; Pre: precision; Sen: sensitivity; F1: F-measure; MCC: Matthews correlation coefficient; ACC: accuracy.

**Table 3:** Comparison of the performance of three classifiers.

Schemes	AUC	Spe	Pre	Sen	F1	MCC	ACC
*k*-nearest neighbor	0.518	0.071	0.331	0.965	0.493	0.073	0.360
Random forest	0.608	0.996	0.964	0.220	0.358	0.387	0.746
Support vector machine	0.879	0.993	0.981	0.773	**0.865**	**0.823**	**0.922**

AUC: area under ROC curve; Spe: specificity; Pre: precision; Sen: sensitivity; F1: F-measure; MCC: Matthews correlation coefficient; ACC: accuracy.

In literature, it is found that some post-translational modification sites are conserved and contained in motifs that are found in different proteins. Therefore, in this work, we investigated whether there exist any characteristic *S*-glutathionylation motifs, which can be used to predict new *S*-glutathionylation proteins and provide insights into the functions related to *S*-glutathionylation sites. For this, we firstly employed PROSITE [16] to scan the flanking sequences of *S*-glutathionylation sites (positive samples) to search for conserved sequence motifs. Then, we obtained the function annotations of these motifs from InterPro database [17] if available. As shown in Table 4, we found four motifs from five positive samples, four of which do not appear in negative samples. According to the function annotations, motif Phosphagen kinase active site signature (PS00112) is possibly related to *S*-glutathionylation due to the close functional connections between phosphorylation and *S*-glutathionylation [4]. Motif Glyceraldehyde 3-phosphate dehydrogenase active site (PS00071) is also likely to be related to *S*-glutathionylation because of the key role of *S*-glutathionylation in cellular oxidative stress regulation. These results indicate that PS00112 and PS00071 are probably catalytic motifs of GSTs, which may help to understand the mechanism of *S*-glutathionylation. With more *S*-glutathionylation proteins coming in the future, we believe more such conserved *S*-glutathionylation motifs will be found.

**Table 4:** Motif analysis results of flanking sequence of *S*-glutathionylation sites.

Protein name	Sequence	Prosite (ID &Pattern)	GO function
Creatine kinase	HLGYVLTCPSNLGTG	PS00112(Phosphagen kinase active site signature): C-P-x(0,1)-[ST]-N-[ILV]-G-T	GO:0016301 kinase activity GO:0016772 transferase activity, transferring phosphorus-containing groups
GAPDH	KIVSNASCTTNCLAP	PS00071(Glyceraldehyde 3-phosphate dehydrogenase active site): [ASV]-S-C-[NT]-T-{S}-x-[LIM]	GO:0016620 oxidoreductase activity, acting on the aldehyde or oxo group of donors, NAD or NADP as acceptor
NF*k*B, p50 and p65	GMRFRYKCEGRSAGS GFRFRYVCEGPSHGG	PS01204(NF*k*B/Rel/dorsal domain signature): F-R-Y-x-C-E-G	GO:0003700 sequence-specific DNA binding transcription factor activity
Papain	NQGSCGSCWAFSAVV	PS00139(Eukaryotic thiol (cysteine) proteases cysteine active site): Q-{V}-x-{DE}-[GE]-{F}-C-[YW]- {DN}-x-[STAGC]-[STAGCV]	GO:0004197 cysteine-type endopeptidase activity

Pattern: Each residue must be separated by − (**minus**). **x** represents any amino acid. **[DE]** means either D or E. **{FWY}** means any amino acid except for F, W and Y. **A(0,2)** means that A appears 0 to 2 times consecutively.

## Discussion

*S*-glutathionylation is a reversible post-translational modification that is important to many biological processes. Identification of protein *S*-glutathionylation sites is essential to the understanding of the functions of proteins. In this work, a framework by employing machine learning approach was developed to predict *S*-glutathionylation sites. With only protein sequence information, our method can obtain promising results with an AUC score of 0.879. Our method can provide biologists candidate *S*-glutathionylation sites for future experimental verification.

In the literature, structural information is also found important to *S*-glutathionylation. For example, low thiol pKa and big surface exposure of cysteine residue were considered to contribute to *S*-glutathionylation. The correlation between accessible surface area (ASA) of cysteine residue and susceptibility of *S*-glutathionylation was derived from the hypothesis that electrostatic interactions were involved in *S*-glutathionylation [18]. However, there are also exceptions. For examples, Ghezzi [19] analyzed the *S*-glutathionylation susceptibility of four Cys residues in cyclophilin A (Cys52, Cys62, Cys115, Cys161) and showed that Cys62 which exposes a smaller surface of its side chains than Cys161 is the target of *S*-glutathionylation while Cys161 is not. Cysteines with thiolate anions at a lowering of pKa value are inclined to have electrostatic interactions with neighboring positively charged amino acid residues. Cytoplasmic proteins that contain this type of cysteine are redox-sensitive and can form a mixed disulfide with GSH. Therefore, a cationic environment renders the protein thiol group highly reactive and particularly susceptible to *S*-glutathionylation [1]. This concept was supported by many examples except ones such as Cys374 of Actin.

In this work, we also investigated the ASA and pKa value of *S*-glutathionylation cysteine and non-*S*-glutathionylation cysteine. We collected the structure information for 39 *S*-glutathionylation proteins by querying the PDB database with both their names and primary sequences. As a result, 36 proteins were retrieved with their 3D structures from PDB database and were kept for further analysis. Subsequently, PROPKA 2.0 [20] and Naccess [21] were respectively used to predict pKa and ASA for these proteins. There are in total 134 samples for the 36 proteins, where 47 samples are positives and 87 are negatives.

**Figure 3:**
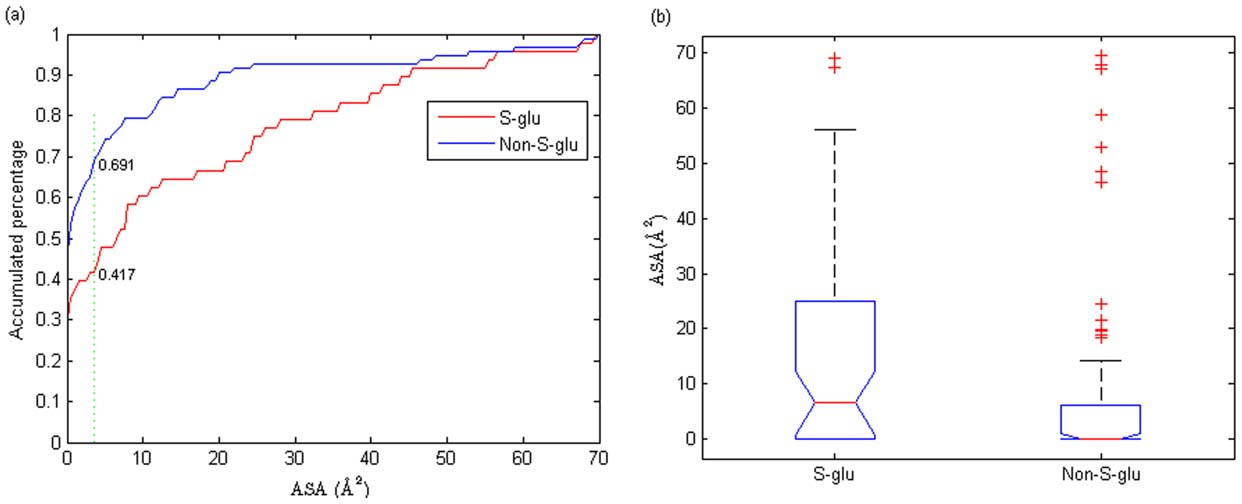
Distributions of thiol ASA values of *S*-glutathionylation cysteine and non-*S*-glutathionylation cysteine. (a) The points on the curve mean the percentage of the samples that have an ASA 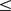 the corresponding ASA value. (b) Box plots of thiol ASA values.

It is found that cysteine thiols with a bigger ASA are more likely to form a mixed disulfide with GSH. In contrast, if cysteine thiols are buried in protein, the formation of disulfide bond will be difficult. Figure 3 shows the distribution of ASA values of *S*-glutathionylation cysteine and non-*S*-glutathionylation cysteine, where the ASA of *S*-glutathionylation cysteine tends to be larger than that of negative samples. In Figure 3a, the biggest difference was obtained between positive samples and negative samples when ASA  = 3.5 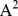, where 69.1% of negative samples have a ASA 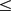
3.5 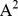 while 41.7% of positive samples with ASA 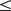
3.5 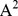. Figure 3b is a box plot of thiol ASA values, in which positive samples have a significant larger ASA than negative samples with 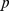-value of 0.0086 based on one-way analysis of variance.

**Figure 4:**
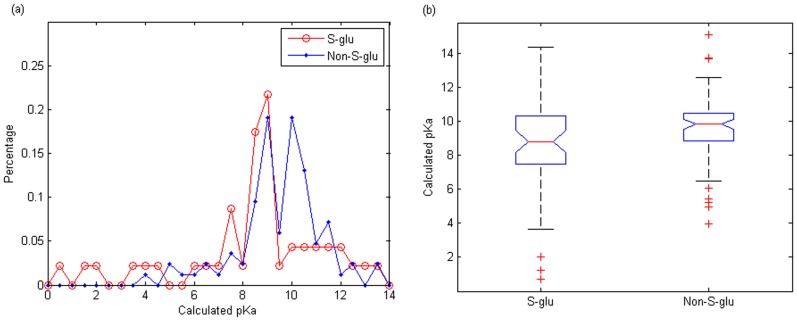
Distributions of thiol pKa values of *S*-glutathionylation cysteine and non-*S*-glutathionylation cysteine. (a) Thiol pKa value distributions. (b) Box plots of thiol pKa values.

Figure 4 shows the distribution of pKa values of *S*-glutathionylation cysteines and non-*S*-glutathionylation cysteines. In our dataset, 15 of 47 (31.9%) positive samples have a pKa value 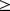
9.00, while 54 of 87 (62.1%) negative samples have a pKa value 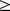
9.00, which is manifested in Figure 4a. The box plot of thiol pKa values in Figure 4b also demonstrates that the pKa median of positive samples is significantly smaller than that of negative ones. From the results shown in Figure 4, we can see that *S*-glutathionylation cysteine tend to have lower pKa value compared to non-*S*-glutathionylation cysteine with a significant 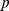-value of 0.0214 based on one-way analysis of variance.

The above results imply that structural information can indeed help to predict *S*-glutathionylation sites. With more structural data available in the future, our method can be easily extended to include the structural information to improve prediction accuracy.

## Materials and Methods

### Datasets

*S*-glutathionylation proteins were collected by employing text mining techniques on published papers retrieved from PubMed. Furthermore, *S*-glutathionylation sites were manually identified and used as positive samples. Cysteines that were not validated to undergo *S*-glutathionylation in experiment were excluded from the database. To construct a supervised classifier for prediction of new *S*-glutathionylation sites, we randomly selected some cysteines except for known *S*-glutathionylation sites from the *S*-glutathionylation protein as negative samples, and the number of negative samples in one *S*-glutathionylation protein is at most three times of that of positive samples in the same protein so that the balance between positive and negative samples is ensured.

In total, the data set contains 39 *S*-glutathionylation proteins, where 75 cysteines undergo reversible *S*-glutathionylation and 158 cysteines that are not susceptible to *S*-glutathionylation. Since the *S*-glutathionylation of cysteine is determined by its position in the protein spatial structure, the flanking sequences of cysteine were taken into account and regarded as the spatial neighborhood of cysteine due to the incompleteness of protein structure data. Finally, the data set consists of 75 positive samples and 158 negative samples, where some cysteines in the negative samples were discarded due to their terminal positions in the protein sequence.

### Feature extraction based on protein sequences

**Figure 5:**
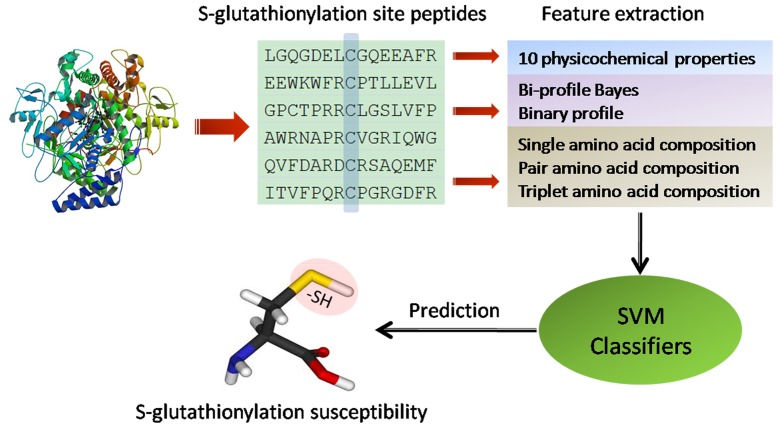
Graphical overview of the method for prediction of protein *S*-glutathionylation sites.

To be used as input for a classifier, each cysteine should be represented as a feature vector. In this work, different feature extraction methods were employed to describe a peptide sequence. Figure 5 shows the flowchart of different feature extraction methods on protein sequences. The details are addressed in following parts.

#### Feature extraction based on physicochemical properties of amino acids

The first type of sequence descriptors considered in feature extraction is the physicochemical features of amino acids, which were taken from the AAIndex database [22]. In this work, 10 widely used physicochemical features were used to encode each sample including number of atoms, number of electrostatic charge, number of potential hydrogen bonds, hydrophobicity, hydrophilicity, propensity, isoelectric point, mass, expected number of contacts within 14 Å sphere, and electron-ion interaction potential. It was found in previous works [23]–[25] that these ten properties correlate well with the interface properties of a protein, which is important for *S*-glutathionylation. For each sample, we summed up the values of all amino acids within the sample and got a 10-dimensional vector. Table S2 describes the values of the ten physicochemical properties for each amino acid.

#### Feature extraction based on sequence profiles

We next consider two sequence profiles Bi-profile Bayes and binary profile. The Bi-profile Bayes method was firstly applied to predict methylation sites and provided significant improvement compared to other methods [12]. In brief, a posterior probability matrix is firstly generated from training datasets according to Bayes' rule and each peptide sequence is then represented by a feature vector based on the posterior probability matrix. In our work, a 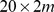 posterior probability matrix 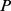 was generated, where 20 denotes the 20 amino acids, 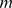 denotes the length of the peptide sequence(15 here), and each element 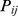 in the matrix denotes the occurrence frequency of the amino acid 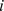 at position 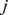 in positive datasets (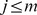) or the occurrence frequency of amino acid 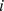 at position 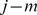 in negative datasets (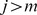). According to this posterior probability matrix 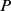, each sample was converted into a 30-dimensional vector.

Binary profile is a simple encoding method that utilizes the position-specific information of each amino acid, and has been successfully used to predict the caspase cleavage sites [26]. In this method, each amino acid is encoded into a 20-dimensional vector, e.g. A(10000000000000000000), C(01000000000000000000), D(00100000000000000000), ..., Y(00000000000000000001). With this encoding, we can represent each sample as a 300-dimensional feature vector.

#### Feature extraction based on amino acid composition

Since all structural and functional information are encoded by amino acids in protein sequences, three different feature extraction approaches were used to describe each sample based on their amino acid compositions, including single amino acid composition, pairwise amino acid composition and triplet-wise amino acid composition.

For the single amino acid composition, the occurrence frequency of each amino acid in the sample was used to encode a sample. For example, a sample will be represented as a 20-dimensional vector if we consider the 20 amino acids in nature. In this work, except for the natural amino acids, the 20 amino acids can be grouped into different clusters based on distinct properties, thereby leading to reduced amino acids. Here, the 3 reduced amino acids and 7 reduced amino acids were used [27], and the reduced amino acids can be found in Table 5. For 3 reduced amino acids, the 20 amino acids were grouped into three classes, i.e. polar, neutral, and hydrophobic. For 7 reduced amino acids, the 20 amino acids were grouped into seven classes, i.e. aliphatic, acid, base, aromatic, amide, small hydroxyl, and sulfur-containing.

**Table 5:** Reduced amino acids based on different properties.

3 classes reduced amino acid	Polar (R,K,E,D,Q,N)
	Neutral (G,A,S,T,P,H,Y)
	Hydrophobic (C,V,L,I,M,F,W)
7 classes reduced amino acid	Aliphatic (A,I,L,V,G,P )
	Acid (D,E)
	Base (H,K,R)
	Aromatic (F,W,Y)
	Amide (N,Q)
	Small hydroxyl (S,T)
	Sulfur-containing (C,M)

For pairwise amino acid composition, all possible combinations of two amino acids were considered and the occurrence frequency of each amino acid pair within the sample was used to describe the samples. As a result, each sample can be represented as a 400-dimensional feature vector. This encoding approach has been widely used in literature [28].

For triplet-wise amino acid composition (ThrAA), all possible combinations of three amino acids were considered and the occurrence frequency of each amino acid triplet within the sample was used to describe the sample. The amino acid triplets was proved to be an effective descriptor of proteins for predicting protein-protein interactions [29] and subcellular localization [30]. In this way, each sample can be described as a 8000-dimensional feature vector. Furthermore, we consider a triplet and its reversed version as the same. For example, ‘ABD’ and ‘DBA’ are treated as the same. Consequently, each sample can be represented as a 4200-dimensional vector with reduced amino acid triplet compositions.

### Model construction

As the values of 10 physicochemical properties are described in different range, each feature value is normalized as following:
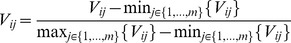
(7)where 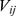 is the value for feature 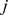 in vector 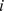, and 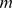 is the number of samples. Since each sample is described with thousands of features, it leads to high computation cost and the noise in the data generally degrades the performance of classifiers. Therefore, it is necessary to reduce the dimensionality with feature selection and remove noise from signals. To find out informative features, the Student's 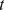-test was utilized to rank the features and the top ranked features were used to construct a classifier. The classifiers built here are support vector machines (SVMs), that was implemented with LIBSVM [31], where the parameters were optimized with 5-fold cross-validation.

## Supporting Information

Table S1 **The effects of different window sizes on SVM performance.**
(DOC)

Table S2 **The values of the ten physicochemical properties for each amino acid.** NA: Number of atoms; NE: Number of electrostatic charge; NP: Number of potential hydrogen bonds; HB: Hydrophobicity; HL: Hydrophilicity; PP: Propensity; IP: Isoelectric points; MA: Mass; EN: Expected number of contacts within 14 Å sphere; EI: Electron-ion interaction potential.
(DOC)
