# Supplementary material for: Prediction of S-Glutathionylation Sites Based on Protein Sequences
Source: PLoS One. 2013 Feb 13;8(2):e55512. doi: 10.1371/journal.pone.0055512 (PMC3572087; doi:10.1371/journal.pone.0055512)
Supplement: Table S1 — The effects of different window sizes on SVM performance. (DOC) [file pone.0055512.s001.doc]

Table S1. The effects of different window sizes on SVM performance.

| Schemes | AUC | Spe | Pre | Sen | F1 | MCC | ACC |
| --- | --- | --- | --- | --- | --- | --- | --- |
| Length=9 | 0.782 | 1.000 | 1.000 | 0.587 | 0.739 | 0.700 | 0.867 |
| Length=11 | 0.803 | 1.000 | 1.000 | 0.627 | 0.770 | 0.730 | 0.880 |
| Length=13 | 0.859 | 0.987 | 0.965 | 0.727 | 0.829 | 0.778 | 0.903 |
| Length=15 | **0.879** | 0.993 | 0.981 | 0.773 | **0.865** | **0.823** | **0.922** |
| Length=17 | 0.859 | 0.978 | 0.939 | 0.734 | 0.824 | 0.766 | 0.899 |
| Length=19 | 0.876 | 0.966 | 0.914 | 0.762 | 0.831 | 0.767 | 0.900 |
| Length=21 | 0.876 | 0.967 | 0.913 | 0.721 | 0.806 | 0.739 | 0.888 |

AUC: area under ROC curve; Spe: specificity; Pre: precision; Sen: sensitivity; F1: F-measure; MCC: Matthews correlation coefficient; ACC: accuracy
