# Supplementary material for: Prediction of S-Glutathionylation Sites Based on Protein Sequences
Source: PLoS One. 2013 Feb 13;8(2):e55512. doi: 10.1371/journal.pone.0055512 (PMC3572087; doi:10.1371/journal.pone.0055512)
Supplement: Table S2 — The values of the ten physicochemical properties for each amino acid. NA: Number of atoms; NE: Number of electrostatic charge; NP: Number of potential hydrogen bonds; HB: Hydrophobicity; HL: Hydrophilicity; PP: Propensity; IP: Isoelectric points; MA: Mass; EN: Expected number of contacts within 14 Å sphere; EI: Electron-ion interaction potential. (DOC) [file pone.0055512.s002.doc]

Table S2. The values of the ten physicochemical properties for each amino acid.

| Residue | NA | NE | NP | HB | HL | PP | IP | MA | EN | EI |
| --- | --- | --- | --- | --- | --- | --- | --- | --- | --- | --- |
| A | 5 | 0 | 2 | 0.25 | 3 | -0.17 | 6.11 | 71.1 | -0.22 | 0.0373 |
| C | 6 | 0 | 2 | 0.04 | -1 | 0.43 | 6.31 | 103.1 | 4.66 | 0.0829 |
| D | 8 | -1 | 4 | -0.72 | 3 | -0.38 | 5.945 | 115.1 | -4.12 | 0.1263 |
| E | 9 | -1 | 4 | -0.62 | 3 | -0.13 | 5.785 | 129.1 | -3.64 | 0.0058 |
| F | 11 | 0 | 2 | 0.61 | -2.5 | 0.82 | 5.755 | 147.2 | 5.27 | 0.0946 |
| G | 4 | 0 | 2 | 0.16 | 0 | -0.07 | 6.065 | 57 | -1.62 | 0.005 |
| H | 10 | 0 | 4 | -0.4 | -0.5 | 0.41 | 5.565 | 137.1 | 1.28 | 0.0242 |
| I | 8 | 0 | 2 | 0.73 | -1.8 | 0.44 | 6.04 | 113.2 | 5.58 | 0 |
| K | 9 | 1 | 2 | -1.1 | 3 | -0.36 | 5.61 | 128.2 | -4.18 | 0.0371 |
| L | 8 | 0 | 2 | 0.53 | -1.8 | 0.4 | 6.035 | 113.2 | 5.01 | 0 |
| M | 8 | 0 | 2 | 0.26 | -1.3 | 0.66 | 5.705 | 131.2 | 3.51 | 0.0823 |
| N | 8 | 0 | 4 | -0.64 | 0.2 | 0.12 | 5.43 | 114.1 | -2.65 | 0.0036 |
| P | 7 | 0 | 2 | -0.07 | 0 | -0.25 | 6.295 | 97.1 | -3.03 | 0.0198 |
| Q | 9 | 0 | 4 | -0.69 | 0.2 | -0.11 | 5.65 | 128.1 | -2.76 | 0.0761 |
| R | 11 | 1 | 4 | -1.76 | -0.5 | 0.27 | 5.405 | 156.2 | -0.93 | 0.0959 |
| S | 6 | 0 | 4 | -0.26 | 0.3 | -0.33 | 5.7 | 87.1 | -2.84 | 0.0829 |
| T | 7 | 0 | 4 | -0.18 | -0.4 | -0.18 | 5.595 | 101.1 | -1.2 | 0.0941 |
| V | 7 | 0 | 2 | 0.54 | -1.5 | 0.27 | 6.015 | 99.1 | 4.45 | 0.0057 |
| W | 14 | 0 | 3 | 0.37 | -3.4 | 0.83 | 5.935 | 186.2 | 5.2 | 0.0548 |
| Y | 12 | 0 | 3 | 0.02 | -2.3 | 0.66 | 5.705 | 163.2 | 2.15 | 0.0516 |

NA: Number of atoms

NE: Number of electrostatic charge

NP: Number of potential hydrogen bonds

HB: Hydrophobicity

HL: Hydrophilicity

PP: Propensity

IP: Isoelectric points

MA: Mass

EN: Expected number of contacts within 14 Å sphere

EI: Electron-ion interaction potential
